# Aquatic Interventions for Children and Adolescents With Autism Spectrum Disorder: A Systematic Review and Meta-Analysis of Motor, Sleep, Executive Function, and Adaptive Measures

**DOI:** 10.7759/cureus.116809

**Published:** 2026-09-23

**Authors:** Hussein M Ziab, Rami Mazbouh, Abdullah Alshami, Kalpana Singh, Abdulqadir J Nashwan

**Affiliations:** 1 Al-Maha Pediatric Specialized Care Center, Al-Wakra Hospital, Hamad Medical Corporation, Doha, QAT; 2 Physiotherapy, Islamic University of Lebanon, Baalback, LBN; 3 Research and Development/Physiotherapy, Health Rehabilitation Integration Research Center, Beirut, LBN; 4 Medical Research, Hamad Medical Corporation, Doha, QAT; 5 Nursing and Midwifery Research, Certified Global Nurse Consultant, Doha, QAT

**Keywords:** adaptive behavior, aquatic therapy, autism spectrum disorder, child, hydrotherapy, motor skills

## Abstract

Children and adolescents with autism spectrum disorder (ASD) frequently face challenges in motor coordination, balance, sensory processing, sleep, physical activity engagement, and social and adaptive functioning. Aquatic interventions can offer a supportive therapeutic setting by combining physical activity, sensory input, motor practice, and opportunities for functional participation. This systematic review and meta-analysis examined the effects of structured aquatic interventions in children and adolescents with ASD on aquatic skills and water safety, sleep, social and adaptive outcomes, motor and balance performance, executive function, biomarkers, quality of life, and participation-related outcomes. A search was conducted in PubMed, CINAHL, Web of Science, Cochrane CENTRAL, and PEDro for studies involving participants aged 0 to 18 years with ASD who underwent structured hydrotherapy, aquatic therapy, swimming-based therapy, or other water-based interventions. A narrative synthesis was carried out for all eligible studies, while random-effects meta-analyses were conducted for controlled studies reporting comparable outcomes.

Nineteen studies, reporting ASD samples totaling 342 participants, were included, and seven of these contributed to the quantitative synthesis. Aquatic skills and water safety produced the most consistently favorable findings. Pooled estimates favored aquatic intervention for sleep (Hedges' g = 1.76, 95% confidence interval (CI): 1.16 to 2.37), motor and balance outcomes (g = 1.49, 95% CI: 0.91 to 2.07), and executive function (g = 0.92, 95% CI: 0.35 to 1.46). Social and adaptive outcomes similarly favored aquatic intervention, though the pooled effect did not reach statistical significance (g = 0.38, 95% CI: −0.17 to 0.94). Since most domain-specific meta-analyses included only two studies, the pooled estimates should be viewed with caution. Overall, aquatic interventions appear to support water safety, motor and balance performance, sleep, and possibly executive function in pediatric ASD, while effects on social and adaptive functioning remain uncertain.

## Introduction and background

Autism spectrum disorder (ASD) is a neurodevelopmental condition marked by persistent differences in social communication and social interaction, along with restricted, repetitive patterns of behavior, interests, or activities [1]. The prevalence of ASD has risen globally, with recent surveillance data showing that ASD affects a substantial and growing proportion of children [2]. While the diagnostic criteria emphasize social communication and restricted or repetitive behaviors, ASD is often associated with a broad range of developmental and functional difficulties, including motor impairment, sensory processing differences, sleep disturbance, reduced physical activity participation, emotional dysregulation, and challenges in adaptive behavior [3,4,5].

Motor difficulties are increasingly seen as clinically important in children with ASD. These can include impairments in postural control, balance, bilateral coordination, praxis (the ability to plan and carry out purposeful movements), gait, object control, motor planning, and overall gross motor skills [6,7]. These difficulties are not merely isolated physical limitations; they can restrict participation in play, peer interaction, and physical activities. Children with ASD often participate less in organized physical activity than their typically developing peers, in part because of motor difficulties, communication challenges, sensory sensitivities, reduced motivation, anxiety, and difficulty managing complex social or rule-based environments [8].

Sleep disturbance is another common and clinically significant concern in ASD. Difficulties with sleep onset, night waking, reduced sleep duration, bedtime resistance, and irregular sleep patterns have been documented in a high proportion of children with ASD and may contribute to daytime behavioral problems, caregiver stress, impaired learning, and reduced quality of life [9,10]. Physical activity has been suggested as a non-pharmacological strategy to support sleep regulation by increasing energy expenditure, improving circadian rhythms, reducing anxiety, and supporting the regulation of physiological arousal [11]. That said, the optimal type, intensity, and delivery context of physical activity for children with ASD remain unclear.

Aquatic intervention has drawn increasing attention as a potentially suitable therapeutic and recreational approach for children with ASD. Water offers unique physical properties that may support movement learning and sensory regulation. Buoyancy reduces the body's effective weight and decreases joint loading, allowing children to practice movements that might be difficult or anxiety-provoking on land. Water resistance provides graded strengthening and endurance demands, while hydrostatic pressure, viscosity, turbulence, and temperature offer continuous tactile and proprioceptive input (sensory information about body position and movement) that may support postural control and arousal regulation [12,13]. These features may make aquatic environments especially suitable for children with ASD who experience balance difficulties, sensory processing differences, low confidence, or reduced tolerance for competitive land-based activities.

Aquatic intervention is also highly relevant from a safety standpoint. Children with ASD may face increased risk of unintentional injury and drowning, particularly due to wandering or elopement behaviors and water attraction [14,15]. As a result, outcomes such as water orientation, breath control, floating, propulsion, safe pool entry and exit, and basic water safety are not only motor or recreational goals, but also functional and potentially life-preserving skills. For families, improved water competency may boost confidence, reduce restrictions around aquatic environments, and support participation in community and family activities.

Beyond water safety and swimming competence, aquatic interventions may plausibly affect broader developmental outcomes. Structured aquatic programs can combine task-specific motor learning, sensory input, predictable routines, therapist-mediated interaction, graded exposure, and enjoyable physical activity. These components may support motor skills, adaptive behavior, social engagement, sleep regulation, and executive function (higher-order cognitive processes involved in goal-directed behavior, including inhibitory control, working memory, and cognitive flexibility). Exercise interventions, more broadly, have been linked to improvements in motor skills, stereotyped behaviors, sleep, and executive function among children and adolescents with ASD, though effects vary by intervention type, dose, outcome measure, and methodological quality [11,16,17]. These findings offer a broader rationale for examining aquatic intervention as a specific, exercise-based, and sensory-enriched therapeutic modality.

Despite this growing interest, the evidence on aquatic therapy in pediatric ASD remains heterogeneous and limited. Thus, a domain-specific synthesis is needed to clarify where aquatic interventions appear most beneficial, where evidence remains uncertain, and which outcomes need further investigation. This systematic review and meta-analysis set out to synthesize the available evidence on structured aquatic interventions for children and adolescents with ASD. Specifically, the review looked at effects on aquatic skills and water safety, sleep, social/behavioral/adaptive outcomes, motor and balance performance, executive function, biomarkers, quality of life, and participation-related outcomes. By combining controlled quantitative evidence with narrative findings from lower-level designs, the review sought to provide a clinically meaningful and methodologically balanced interpretation of the role of aquatic interventions in ASD rehabilitation.

## Review

Methods

Study Design

This study was a systematic review with narrative synthesis and random-effects meta-analysis. The review brought together the available evidence on aquatic interventions for children and adolescents with ASD. Narrative synthesis was used to summarize findings from all eligible studies, including randomized controlled trials, controlled non-randomized studies, quasi-experimental studies, single-group pre-post studies, single-case designs, and mixed-methods studies. A meta-analysis was performed on controlled studies that had reported sufficiently comparable quantitative data.

The review was carried out and reported following the Preferred Reporting Items for Systematic Reviews and Meta-Analyses (PRISMA) framework. A review protocol was drafted in July 2025, before study selection and data synthesis. The protocol was registered prospectively in PROSPERO under registration number CRD420251087912.

Review Question and Eligibility Criteria

The review question was structured according to the Population-Intervention-Comparator-Outcome (PICO) framework. The population comprised children and adolescents aged 0-18 years diagnosed with ASD. Eligible interventions included structured hydrotherapy, aquatic therapy, swimming-based therapy, or other water-based interventions. Comparators included usual care, no intervention, conventional therapy, or alternative active interventions such as land-based therapy. Outcomes of interest included motor performance, gross motor skills, balance, adaptive behavior, sensory and behavioral responses, social communication, executive function, quality of life, aquatic skills, and related physiological markers.

Studies were eligible if they were full-text articles published in peer-reviewed journals, written in English, and involved children with ASD receiving a structured aquatic intervention with either pre-post assessment or comparison with a control or alternative intervention group. To ensure comprehensive coverage of the literature, a broad range of interventional designs was considered, including randomized controlled trials, controlled trials, quasi-experimental studies, pilot studies, single-group pre-post studies, mixed-methods studies, and single-case designs. Reviews, editorials, non-translated publications, studies involving adults or non-ASD populations, and studies reporting outcomes outside the review scope were excluded.

Data Sources and Search Strategy

A comprehensive literature search for studies published between January 2000 and December 2025 was conducted in PubMed, CINAHL, Web of Science, CENTRAL, and PEDro. Database searching was supplemented by manual checking of the reference lists of included studies. Search strategies were adapted to the indexing system and syntax of each database. The complete database-specific search strategies and applied limits are provided in Table 1.

**Table 1:** Database-specific search strategies ASD: autism spectrum disorder; CINAHL: Cumulative Index to Nursing and Allied Health Literature; CENTRAL: Cochrane Central Register of Controlled Trials; MeSH: Medical Subject Headings; PEDro: Physiotherapy Evidence Database; TS: Topic; MH: CINAHL Subject Heading

Database	Search field/indexing	Search strategy
PubMed	MeSH and Title/Abstract	("Hydrotherapy"[MeSH] OR "Aquatic Therapy"[Title/Abstract] OR "Water-based therapy"[Title/Abstract] OR "Water exercise"[Title/Abstract]) AND ("Autism Spectrum Disorder"[MeSH] OR "Autism"[Title/Abstract] OR "ASD"[Title/Abstract]) AND ("Child"[MeSH] OR "Children"[Title/Abstract] OR "Pediatric"[Title/Abstract])
CINAHL via EBSCOhost	CINAHL Subject Headings (MH) and free-text terms	(MH "Hydrotherapy" OR "aquatic therapy" OR "water-based therapy" OR "water exercise") AND (MH "Autism Spectrum Disorder" OR "autism" OR "ASD") AND (MH "Child" OR "children" OR "pediatric")
Web of Science Core Collection	Topic search (TS=), covering title, abstract, and keywords	TS=("hydrotherapy" OR "aquatic therapy" OR "water-based therapy" OR "water exercise") AND TS=("autism" OR "autism spectrum disorder" OR "ASD") AND TS=("child" OR "children" OR "pediatric")
CENTRAL	Keyword search	(hydrotherapy OR "aquatic therapy" OR "water-based therapy" OR "water exercise") AND (autism OR "autism spectrum disorder" OR ASD) AND (child OR children OR pediatric)
PEDro	Advanced Search; free-text Abstract and Title field combined with PEDro indexed fields	Abstract & Title = "Autism Spectrum Disorder" combined using AND with the Therapy, Problem, and Subdiscipline fields selected in the PEDro Advanced Search interface. The archived search URL recorded the selections as therapy=VL01382, problem=VL01375, and subdiscipline=VL01360.

All retrieved records were imported into the reference management workflow (Microsoft Excel) and screened for duplicates. Title and abstract screening were performed by two reviewers (HZ and RM) before full-text review. Studies judged potentially eligible or with uncertain eligibility underwent a full-text assessment. Disagreements regarding inclusion were resolved through discussion and, when required, consultation with an additional reviewer.

Data Extraction

Data were extracted by one reviewer (HZ) into a structured screening and data-extraction table (Tables 2-3). For each study, the following information was recorded: title, authors, year of publication, study design, sample size, diagnosis, age, sex distribution, intervention characteristics, control/comparison condition, outcome measures, and main findings. Intervention details extracted included duration, weekly frequency, session length, delivery format, and therapeutic approach, where reported. Outcomes were subsequently grouped into conceptually related domains, including social/adaptive outcomes, sleep outcomes, motor/balance outcomes, aquatic skills and water safety, executive function, and biomarker or quality-of-life outcomes.

**Table 2:** Raw data and effect sizes used in the quantitative synthesis CSHQ: Children’s Sleep Habits Questionnaire; SD: standard deviation; SRS: Social Responsiveness Scale; VABS: Vineland Adaptive Behavior Scales; WCST: Wisconsin Card Sorting Test

Domain	Outcome	Study	Intervention			Control			Direction	EZ
			N	Mean	SD	N	Mean	SD		
Social/adaptive	VABS composite scale	Caputo et al., 2018 [17]	13	66.15	11.39	13	61.00	7.25	Higher = better	0.52
	SRS	Zanobini and Solari, 2019 [18]	13	91.31	24.31	12	97.75	27.50	Lower = better	0.24
Sleep	CSHQ	Adib et al., 2019 [19]	10	57.80	6.76	10	69.30	1.82	Lower = better	2.22
		Ansari et al., 2021 (sleep) [20]	20	51.85	4.65	20	60.80	6.32	Lower = better	1.58
Motor/balance	Dynamic balance	Ansari et al., 2021 (kata) [21]	10	9.60	1.34	10	6.70	1.59	Higher = better	1.89
		Faraji et al., 2023 [22]	20	2.80	1.80	20	0.98	0.60	Higher = better	1.33
Executive function	WCST correct responses	Faraji et al., 2023 [22]	20	38.70	7.90	20	32.40	9.60	Higher = better	0.70
	Flanker accuracy	Zhao et al., 2024 [23]	16	—	—	14	—	—	Higher = better	1.23*

**Table 3:** Characteristics of studies included in the systematic review ASD: autism spectrum disorder; BDNF: brain-derived neurotrophic factor; BOTMP-2: Bruininks-Oseretsky Test of Motor Proficiency, Second Edition; CARS: Childhood Autism Rating Scale; CSHQ: Children’s Sleep Habits Questionnaire; OT: occupational therapy; PedsQL: Pediatric Quality of Life Inventory; PSPCSA: Pictorial Scale of Perceived Competence and Social Acceptance; RCT: randomized controlled trial; SRS: Social Responsiveness Scale; VABS: Vineland Adaptive Behavior Scales; WCST: Wisconsin Card Sorting Test; WOTA: Water Orientation Test Alyn

Study	Design/sample	Age	Aquatic intervention	Comparator	Intervention dose	Main outcomes	Main findings
Yilmaz et al., 2004 [24]	Case study; n = 1	9 years	Halliwick-based swimming/water-orientation training	None	10 week; 3×/wk; 60 min	Water orientation; physical fitness; stereotypic behavior	Improved water orientation and physical performance; stereotypic movements decreased
Pan, 2010 [25]	Controlled crossover study; n = 16 boys	6–9 years	Water Exercise Swimming Program (WESP), Halliwick-based	Delayed-treatment/control phase	10-wk intervention phases; 2×/wk; 90 min	Aquatic skills; social behavior	Aquatic skills improved after WESP; selected social-behavior improvements were also observed
Fragala-Pinkham et al., 2011 [13]	Non-randomized controlled pilot; n = 12 (7 intervention, 5 control)	Children with ASD	Group swimming and aquatic exercise	Control	14 wk; 2×/wk; 40 min	Swimming skills; cardiorespiratory and muscular endurance; mobility; satisfaction	No significant between-group fitness changes; within-group swimming skills improved; high satisfaction and feasibility
Pan, 2011 [26]	Controlled single-blinded crossover study; 15 children with ASD + 15 siblings	7–12 years	Structured aquatic program	Delayed-treatment/crossover comparison	14-wk phases	Aquatic skills; physical fitness	Improved aquatic skills and several physical-fitness components following aquatic intervention
Oriel et al., 2014 [30]	A-B-A withdrawal pilot; n = 8	Children with ASD	Aquatic exercise	Baseline/withdrawal phases	Each phase 4 wk; intervention 2×/wk; 60 min	Sleep latency; night waking; sleep duration	Reduced sleep latency and increased sleep duration during aquatic intervention, with some regression after withdrawal
Yanardag et al., 2015 [27]	Multiple-probe single-case design; n = 3	6 years	One-to-one aquatic skill training using most-to-least prompting	Baseline/probe phases	3×/wk	Advanced aquatic movement/exploration skills	All children acquired targeted aquatic skills, with maintenance and generalization demonstrated
Alaniz et al., 2017 [28]	Pilot pre-post study; n = 7	Children with mild-to-severe ASD	Aquatic group therapy/water-safety training	None	8 h total intervention	Aquatic Skills Checklist; social skills	Significant improvement in water-safety skills; social-skills scores did not significantly change
Mische Lawson and Little, 2017 [31]	Pre-post feasibility study; n = 10	5–12 years	Sensory Enhanced Aquatics swimming program	None	8 wk	Sleep disturbance; sensory processing	Feasible and highly acceptable; sleep responses varied according to sensory characteristics and autism severity
Caputo et al., 2018 [17]	Non-randomized controlled study; n = 26 (13 intervention, 13 control in quantitative comparison)	Children with ASD	Multisystem Aquatic Therapy: emotional adaptation, swimming adaptation, social integration	Control/usual treatment	10 mo	VABS; CARS; behavioral/emotional functioning; swimming skills	Improved functional adaptation, emotional responses, adaptation to change, activity level, and swimming skills
Adib et al., 2019 [19]	Randomized pre-post controlled study; n = 20 (10/10)	8–14 years	Aquatic exercise	No aquatic intervention	10 wk; 2×/wk; 60 min	CSHQ; stereotypic behavior	Significant improvements in total sleep problems, several sleep domains, and stereotypic behaviors
Zanobini and Solari, 2019 [18]	Matched controlled study; n = 25 (13 intervention, 12 control)	3–8 years	Acqua Mediatrice di Comunicazione swimming program	Matched comparison group receiving other activities/therapies	12 wk; 1×/wk; 30 min	SRS; autistic behaviors; aquatic skills	Improved relational/social skills and aquatic abilities; some benefits persisted at follow-up
Mills et al., 2020 [32]	Randomized crossover-controlled pilot; n=8	6–12 years	Hydrotherapy	Delayed/crossover condition	8-wk study; 4-wk intervention phase	Child Behavior Checklist; mental-health/well-being behaviors	Improvements reported in internalizing, anxiety/depression, attention, thought problems, and total behavioral problems
Ansari et al., 2021 – sleep [20]	RCT; n = 40 (20 aquatic, 20 control)	6–14 years	Water-based aquatic exercise	No intervention	10 wk; 2×/wk; 60 min	CSHQ; IL-1β; TNF-α	Improved sleep quality and reduced IL-1β and TNF-α compared with control
Ansari et al., 2021 – balance [21]	Three-group RCT; n = 30	8–14 years	Aquatic training	Kata training and control	10 wk; 2×/wk; 60 min	Static and dynamic balance	Both aquatic and kata exercise improved balance; improvement was greater for some outcomes in the kata group.
Güeita-Rodríguez et al., 2021 [33]	Mixed-methods pre-post intervention; n = 6	6–12 years	Water Specific Therapy–Halliwick	Pre-post; no separate control	Continued structured aquatic therapy	PedsQL; PSPCSA; WOTA-1; qualitative outcomes	Improved physical competence and aquatic functioning; favorable changes in school functioning, participation, and family-reported experiences
Walsh et al., 2021 [29]	Mixed-methods pilot cohort; n = 8	3–7 years	Individualized aquatic OT/adaptive swimming	None	10 wk; 1×/wk; 60 min	WOTA-2; Goal Attainment Scaling; caregiver experience	Children improved aquatic skills and individualized goals; high caregiver satisfaction
Faraji et al., 2023 [22]	Quasi-experimental controlled study; n = 40 (20/20)	7–10 years	Response-oriented aquatic exercise	Control	Structured aquatic program	BOTMP-2; WCST; static/dynamic balance	Improved motor skills, static and dynamic balance, and several executive-function indices
Zhao et al., 2024 [23]	Controlled intervention study; n = 30	Children with ASD	Aquatic exercise	Supervised free activities	12 wk	Executive function; Flanker-related outcomes; BDNF	Improved inhibitory control and cognitive flexibility and increased BDNF; working memory did not significantly improve
Kemp et al., 2024 [40]	Single-blind randomized waitlist-controlled trial; n = 37	5–9 years	AquOTic: individualized OT-based water-competency intervention using task-specific practice, play, sensory support, positive reinforcement, and modified Halliwick principles	Waitlist control	10 wk; 1×/wk	WOTA-1; WOTA-2; Swim Skills Checklist; water competency	Significant improvements in WOTA-1, WOTA-2, swimming skills, and several components of water competency after AquOTic

Risk of Bias

Formal risk-of-bias assessment was restricted to studies contributing to the quantitative synthesis [17,18,19,20,21,22,23]. Randomized controlled trials were assessed using the revised Cochrane Risk of Bias tool (RoB 2), whereas non-randomized controlled and quasi-experimental studies contributing to the quantitative synthesis were assessed using the Risk of Bias in Non-randomized Studies of Interventions tool (ROBINS-I) (Table 4). Studies included only in the narrative synthesis were used as supportive evidence, and their methodological limitations were considered qualitatively when interpreting the findings.

**Table 4:** Risk-of-bias tool selection, outcome sources, and effect-size coding used in the risk-of-bias stratified forest plots This table presents outcome-specific risk-of-bias judgments for studies contributing to the quantitative synthesis. Randomized controlled trials were assessed using RoB 2, whereas non-randomized or quasi-experimental controlled studies were assessed using ROBINS-I. The forest plots are stratified by overall risk-of-bias judgment and use the same color logic as the RoB 2 example figure: green (+) = low risk, yellow (?) = some concerns/moderate risk, and red (-) = high/serious risk RoB 2: Cochrane risk-of-bias tool for randomized trials; ROBINS-I: Risk of Bias in Non-randomized Studies - of Interventions

Tool	Study	Outcome	Direction coding	Effect size	Main reason for overall judgment
RoB 2	Adib et al., 2019 [19]	CSHQ total score	Lower CSHQ scores indicate better sleep	Hedges’ g = 2.22; 95% CI: 1.09 to 3.36	The outcome was parent-reported sleep. Because allocation was visible and reporter/assessor blinding was not clearly described, outcome measurement was vulnerable to expectation effects
	Ansari et al., 2021 (sleep) [20]	CSHQ total score	Lower CSHQ scores indicate better sleep	Hedges’ g = 1.58; 95% CI: 0.87 to 2.30	The intervention was visible, and reporter blinding was not clearly reported, creating great concern for measurement bias in the parent-reported sleep outcome
	Ansari et al., 2021 (kata) [21]	Dynamic balance score	Higher dynamic-balance scores indicate better balance	Hedges’ g = 1.89; 95% CI: 0.82 to 2.96	The outcome was performance-based and less subjective than questionnaire outcomes; however, participant/provider blinding was not feasible, and assessor blinding was not clearly reported
	Zhao et al., 2024 [23]	Flanker accuracy	Higher accuracy indicates better executive-function performance	Hedges’ g = 1.23; 95% CI: 0.45 to 2.02	The selected executive-function outcome was relatively objective, but the effect size was derived from an adjusted estimate, and the study had some attrition/reporting concerns
ROBINS-I	Caputo et al., 2018 [17]	Vineland Adaptive Behavior Scales (VABS) composite scale	Higher VABS scores indicate better adaptive functioning	Hedges’ g = 0.52; 95% CI: -0.26 to 1.31	Allocation appeared linked to territorial or geographical feasibility, creating serious concerns about confounding and participant selection
	Zanobini and Solari, 2019 [18]	Social Responsiveness Scale (SRS) total score	Lower SRS scores indicate fewer social-communication difficulties	Hedges’ g = 0.24; 95% CI: -0.55 to 1.03	The matched non-randomized design and incomplete comparative follow-up reporting raised serious concerns about selection bias and confounding
	Faraji et al., 2023 [22]	Dynamic balance score	Higher dynamic-balance scores indicate better balance	Hedges’ g = 1.33; 95% CI: 0.64 to 2.02	Although allocation procedures were described, the quasi-experimental design and recruitment from multiple zones left residual concerns about confounding and implementation

For randomized trials, the RoB 2 assessment considered bias arising from the randomization process, deviations from intended interventions, missing outcome data, measurement of outcomes, and selection of reported results. For non-randomized controlled studies, the ROBINS-I assessment assessed bias due to confounding, participant selection, classification of interventions, deviations from intended interventions, missing outcome data, measurement of outcomes, and selection of the reported result.

Because aquatic and exercise-based interventions are difficult to blind, the lack of blinding of participants and intervention providers was interpreted primarily as a potential source of performance bias rather than as an automatic indication of high detection bias. Detection bias was considered separately according to whether outcome assessment could reasonably have been blinded, whether outcomes were objective or assessor-dependent, and whether the outcome relied on parent-, caregiver-, or therapist-reported measures. Greater concern was assigned when outcome assessors were not blinded, and the outcome measure was subjective or susceptible to expectancy effects.

Risk-of-bias judgments were summarized narratively and presented separately for randomized and non-randomized controlled studies contributing to the quantitative synthesis. Methodological limitations of studies included only in the narrative synthesis were considered qualitatively when interpreting their findings.

Synthesis Strategy and Statistical Analysis

A two-stage synthesis approach was employed. First, all included studies were summarized narratively according to study design and outcome domain. Second, a quantitative synthesis was conducted of controlled studies with comparable outcomes, in which at least two studies contributed data within the same domain. Outcomes were selected using a predefined hierarchy prioritizing primary outcomes, commonly reported measures, validated instruments, and immediate post-intervention time points to minimize selection bias. When multiple eligible outcomes were available within the same domain, one outcome was selected according to this hierarchy to avoid double-counting participants within a meta-analysis.

Domain-specific meta-analyses were performed for social/adaptive function, sleep, motor/balance, and executive-function outcomes using a random-effects model, with between-study variance (τ²) estimated using the DerSimonian-Laird method, to account for clinical and methodological heterogeneity. For studies with available raw group data, effect sizes were calculated as Hedges’ g with 95% confidence intervals (CIs) using post-intervention means and standard deviations. Hedges’ g was used as the standardized mean difference to account for variation in outcome measures and small sample sizes.

The standardized effect size for Flanker accuracy was approximated from the reported adjusted mean difference and 95% CI. The standard error (SE) was calculated as SE = (upper CI − lower CI) / (2 × 1.96), and the t statistic was then calculated as the adjusted mean difference divided by the SE. This value was then converted to Cohen’s d using group sample sizes and subsequently corrected to Hedges’ g using the small-sample correction factor. Because this effect size was derived from an adjusted estimate rather than raw post-intervention means and standard deviations, it was interpreted cautiously.

Statistical heterogeneity was assessed using Cochran’s Q, I², and τ² statistics, and sensitivity analyses included leave-one-out analysis, restriction to randomized controlled trials, and exclusion of studies with approximated estimates. Statistical analyses were conducted using Microsoft Excel, and a two-tailed p-value < 0.05 was considered statistically significant. Assessment of publication bias and small-study effects was not conducted formally because the number of studies included in each domain-specific meta-analysis was limited (fewer than 10).

Results

Study Selection and Characteristics

The literature search identified 101 records. After removing 31 duplicates, 70 records remained for screening. Following title and abstract screening, 8 records were excluded. A total of 62 full-text articles were assessed for eligibility. Of these, 43 articles were excluded: outside scope (n = 13), ineligible study design (n = 3), study proposal only (n = 3), participants aged >18 years (n = 2), no relevant outcomes (n = 2), full text unavailable (n = 1), inadequate sample description/data (n = 1), and other reasons (n = 18). Ultimately, 19 studies met the inclusion criteria and were retained in the final review. Out of them, seven controlled studies contributed to one or more quantitative analyses; domain-specific meta-analyses were performed where at least two studies assessed comparable constructs, whereas the remaining 12 studies were included in the narrative synthesis only. The 19 included studies represented 342 participants with ASD and were published between 2004 and 2024 (Figure 1).

**Figure 1:**
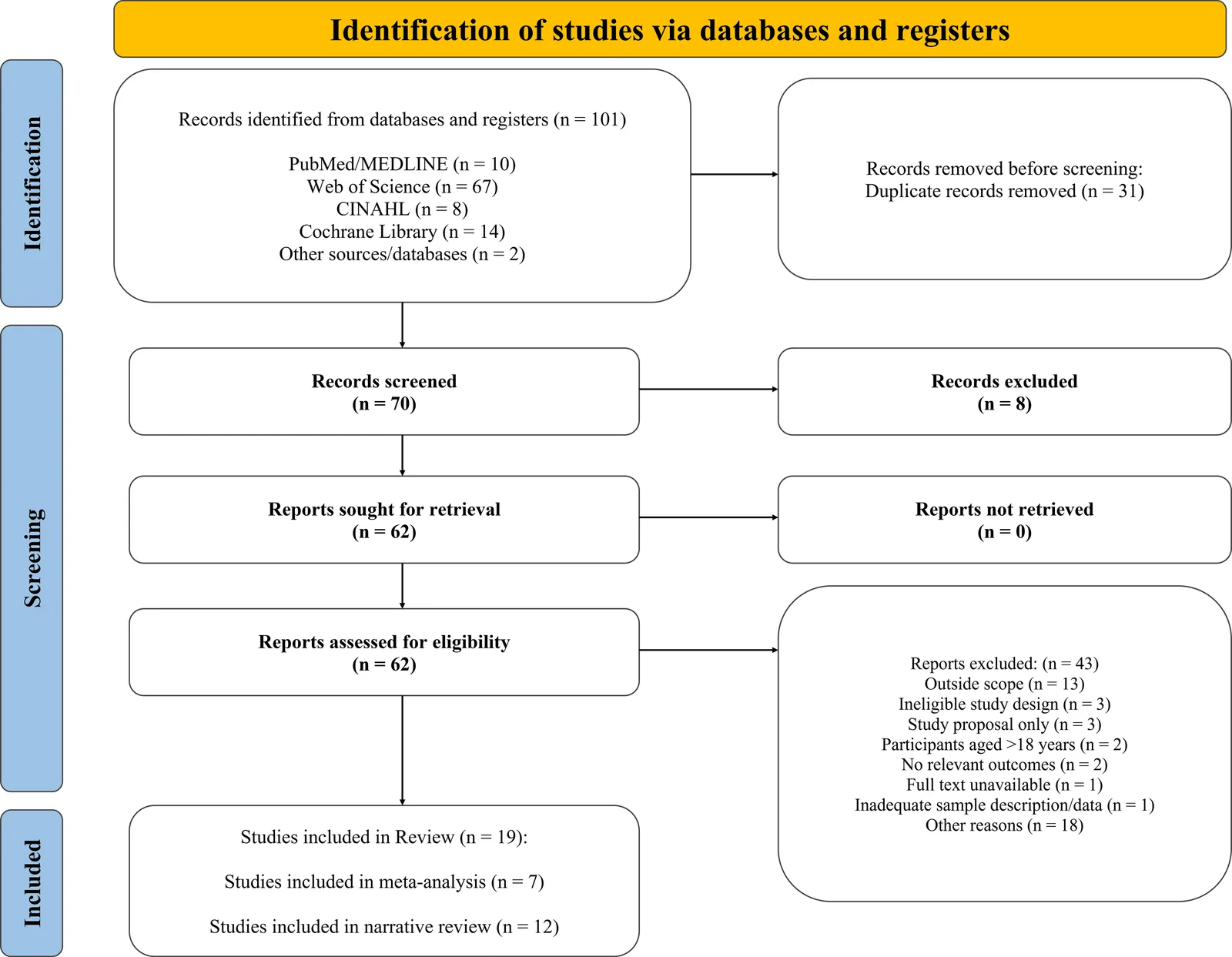
PRISMA flow diagram of study selection PRISMA: Preferred Reporting Items for Systematic reviews and Meta-Analyses

The evidence was methodologically and clinically heterogeneous. Study designs included randomized controlled trials, controlled trials, quasi-experimental studies, crossover-controlled trials, withdrawal designs, single-group pre-post studies, single-case and multiple-probe designs, and mixed-methods studies. Intervention protocols also varied substantially and included Halliwick-based swimming training (a structured approach that uses water adaptation and progressively challenging aquatic movement activities), multisystem aquatic therapy, aquatic exercise, swimming instruction, sensory-enhanced aquatics, aquatic occupational therapy, and adaptive swimming. Intervention duration ranged from four weeks to 10 months, with frequencies from once to three times per week and session lengths generally 30 to 60 minutes. Comparator conditions included no intervention, usual or standard therapy, psychoeducational or sports activities, delayed-treatment phases, free play, and active exercise comparators such as kata training.

Outcomes spanned aquatic readiness and water safety, physical fitness, balance, motor skills, stereotypic behaviors, sleep, adaptive behavior, social responsiveness, executive function, quality of life, participation-related outcomes, and selected biological markers. Because of this variability, all included studies were synthesized narratively, while quantitative synthesis was restricted to controlled studies with extractable data in comparable outcome domains.

Narrative Synthesis

The narrative synthesis was structured by outcome domain and study design. Controlled studies were given greater interpretive weight, while single-case, pre-post, crossover, and mixed-methods studies were used as supportive evidence. Where available, findings are summarized according to study design and direction of effect; detailed pooled effect estimates are presented in the quantitative synthesis.

Aquatic skills and water safety: Aquatic skills and water safety showed the most consistent improvements across study designs. Controlled, pre-post, single-case, and mixed-methods studies reported improvements in aquatic readiness, water orientation, aquatic adjustment, propulsion, breath control, face submersion, object retrieval, individualized swimming goals, and water safety [17,18,24,25,26,27,28,29]. Improvement magnitude varied across aquatic-skill measures, but the overall direction was consistently favorable.

Sleep outcomes: Controlled evidence showed a favorable effect of aquatic intervention on sleep. Adib et al. (2019) and Ansari et al. (2021) [19,20] both used the Children’s Sleep Habits Questionnaire and contributed to the sleep meta-analysis presented in Section 3.3.1.

Lower-level evidence was generally favorable but more variable. Oriel et al. (2014) [30] reported reduced sleep latency and increased sleep duration during the intervention phase, with partial regression after withdrawal, whereas sensory-enhanced aquatic studies reported mixed sleep responses [31]. Overall, sleep outcomes favored aquatic intervention, but interpretation remains cautious because controlled evidence came from only two studies.

Social, behavioral, and adaptive outcomes: Controlled and quasi-controlled evidence showed a favorable but less certain pattern for social, behavioral, and adaptive outcomes. Caputo et al. (2018) [17] and Zanobini and Solari (2019) [18] reported improvements in adaptive behavior, social relating, self-help skills, and autistic mannerisms. These studies contributed to the social/adaptive meta-analysis presented in Section 3.3.1.

Lower-level evidence also suggested possible benefits, but findings were not uniform across studies. Pre-post, crossover, and mixed-methods studies supported possible improvements in social communication, motivation, emotional regulation, participation, and behavioral difficulties. Mills et al. (2020) [32], for example, reported improvements in several behaviors related to mental health and well-being following hydrotherapy.

Motor, balance, and physical fitness: Motor, balance, and physical fitness outcomes were consistently favorable across controlled and lower-level designs. Controlled studies reported improvements in static and dynamic balance, as well as motor skills. Supportive single-case and crossover evidence also showed improvements in balance, agility, flexibility, strength, endurance, aerobic capacity, and water orientation. Overall, this domain showed a clear favorable pattern, although small sample sizes and heterogeneous measures limited the evidence. The corresponding quantitative synthesis is presented in Section 3.3.1.

Executive function: Executive function was assessed in two controlled studies. Faraji et al. (2023) [22] reported improvements in Wisconsin Card Sorting Test indices, while Zhao et al. (2024) [23] reported improvements in Flanker accuracy and related executive-function outcomes. These studies contributed to the executive-function meta-analysis presented in Section 3.3.1. The evidence remains preliminary because only two studies addressed this domain.

Biomarkers and quality-of-life findings: Biomarker and quality-of-life outcomes were reported in a few studies and were not suitable for meta-analysis. Ansari et al. (2021) reported reductions in inflammatory biomarkers alongside sleep improvement, while Zhao et al. (2024) reported increased brain-derived neurotrophic factor alongside executive-function gains [20,23]. Güeita-Rodríguez et al. (2021) and Walsh et al. (2021) reported improvements in participation-related outcomes, quality of life, enjoyment, confidence, and goal attainment [29,33]. These findings are promising but exploratory and should be interpreted as hypothesis-generating.

Benefits extended beyond standardized clinical outcomes. Several studies reported improvements in participation-related outcomes, including individualized aquatic goals, swimming proficiency, water safety, enjoyment, confidence, and caregiver-reported participation. These outcomes were clinically meaningful but not pooled due to heterogeneity in measures and study designs.

Quantitative Synthesis

Domain-specific meta-analyses: The primary quantitative synthesis was conducted using domain-specific meta-analyses rather than a single overall pooled model, because the controlled studies assessed conceptually distinct outcomes. Four domains had at least two controlled studies with extractable quantitative data: social/adaptive outcomes, sleep outcomes, motor/balance outcomes, and executive-function outcomes (Figure 2 and Table 3).

**Figure 2:**
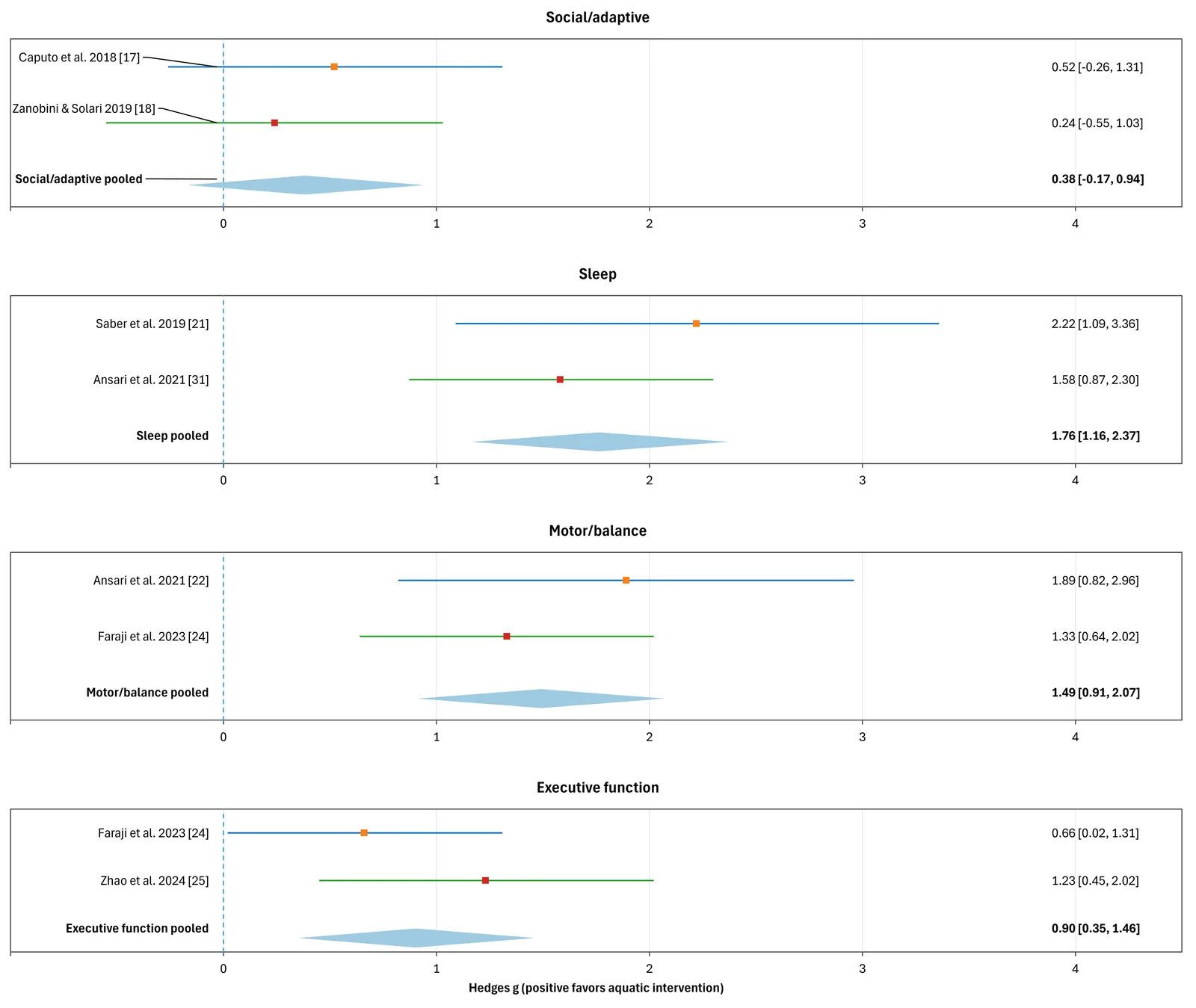
Domain-specific meta-analyses

For social/adaptive outcomes, the pooled estimate favored aquatic intervention but did not reach statistical significance (Hedges’ g = 0.38, 95% CI: -0.17 to 0.94; I² = 0.0%), suggesting a favorable but uncertain effect.

For sleep outcomes, the pooled estimate showed a large effect favoring aquatic intervention (Hedges’ g = 1.76, 95% CI: 1.16 to 2.37; I² = 0.0%). Both studies used the Children’s Sleep Habits Questionnaire, and effect directions were coded so that reductions in sleep-problem scores favored aquatic intervention.

For motor/balance outcomes, the pooled estimate also showed a large effect favoring aquatic intervention (Hedges’ g = 1.49, 95% CI: 0.91 to 2.07; I² = 0.0%). The selected outcomes reflected dynamic balance performance, with higher scores indicating favorable performance.

For executive-function outcomes, the pooled estimate favored aquatic intervention (Hedges’ g = 0.92, 95% CI: 0.35 to 1.46; I² = 5.6%). This model included one effect calculated from raw post-intervention means and standard deviations and one approximate standardized adjusted effect derived from the adjusted mean difference and 95% CI reported by Zhao et al. (2024).

Because these domain-specific models included only two studies (k = 2), the statistical power to detect heterogeneity was low, and the I² estimates may be biased downward toward 0%. Accordingly, I² values close to 0% should not be interpreted as evidence of true homogeneity. These pooled estimates should therefore be regarded as preliminary and interpreted alongside the narrative synthesis rather than as definitive estimates of treatment effect.

Sensitivity and supplementary analyses: Sensitivity analyses were conducted for the supplementary omnibus analysis to examine the stability of the quantitative findings under alternative assumptions. Exclusion of clearly non-randomized controlled studies yielded a significant pooled effect of similar magnitude (g = 1.41, 95% CI: 0.86 to 1.97; I² = 50.1%). Restriction to randomized studies only produced a larger pooled effect with no observed heterogeneity (g = 1.62, 95% CI: 1.18 to 2.06; I² = 0.0%). Exclusion of Zhao et al. (2024), for which the selected effect size was derived from adjusted estimates, did not materially alter the overall result (g = 1.24, 95% CI: 0.65 to 1.82; I² = 65.4%). Leave-one-out analysis further showed that omitting any single study did not change the direction of the effect, indicating that the pooled estimate was not driven by any individual trial (Figure 3).

**Figure 3:**
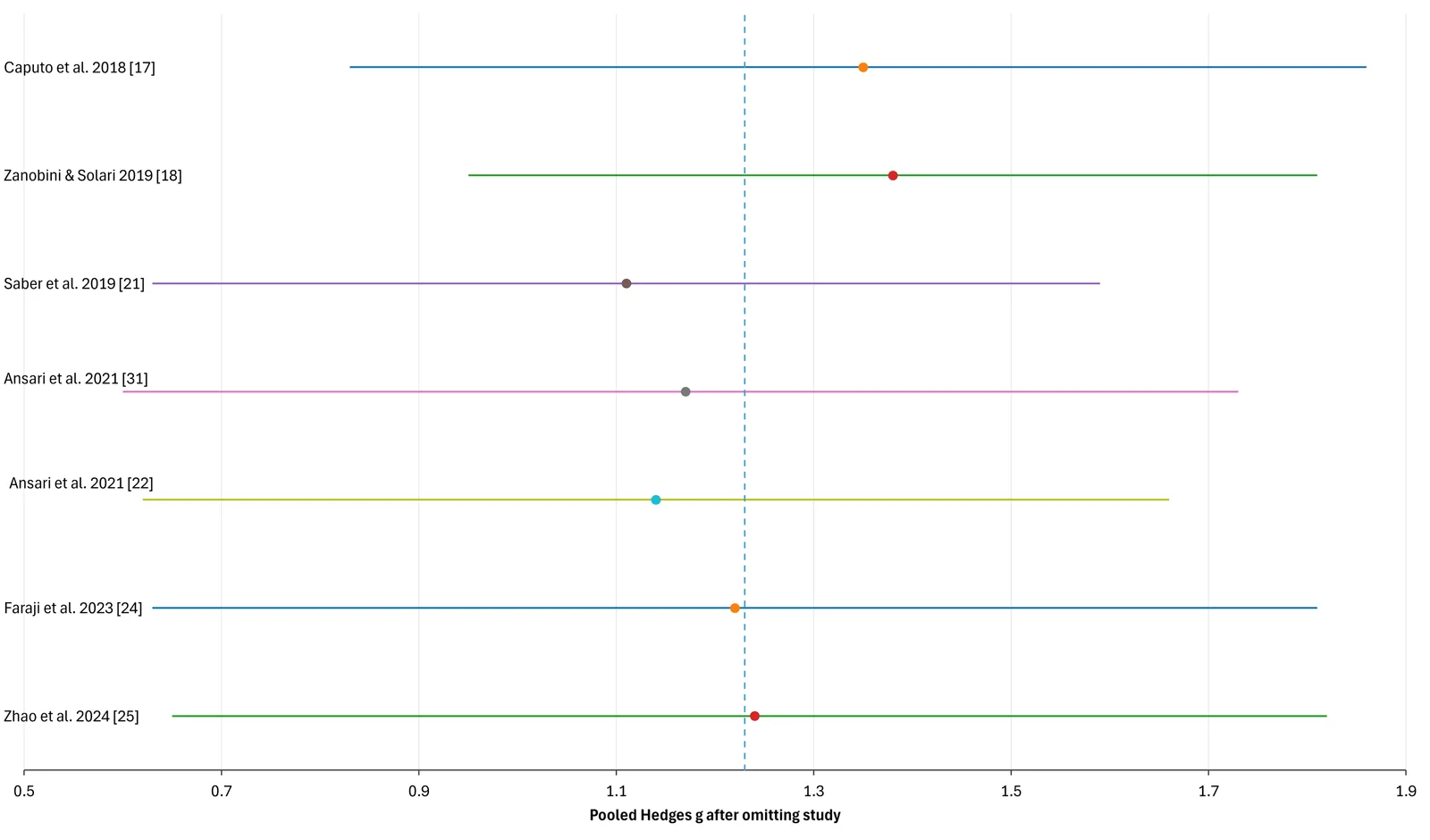
Sensitivity analysis

The exploratory analysis, including one selected outcome from each controlled study, favored aquatic intervention (Table 3); however, this was not treated as a primary result because it combined conceptually distinct constructs, including social/adaptive function, sleep, motor/balance performance, and executive function.

Exploratory assessment of small-study effects: Formal statistical tests for funnel-plot asymmetry were not performed because only seven studies contributed to the quantitative synthesis, making such tests unreliable. A funnel plot was generated only for exploratory visual inspection and is provided in the supplementary material; small-study effects and selective reporting could not be excluded.

Risk of Bias

Randomized controlled trials: Four randomized controlled trials contributing to the quantitative synthesis were assessed using the Cochrane Risk of Bias 2 (RoB 2) tool. The primary methodological concern across studies was deviations from intended interventions, largely due to the impracticality of blinding participants, caregivers, and intervention providers in aquatic or exercise-based settings, which introduced potential performance bias. Detection bias was considered separately and was judged to be of greater concern in studies relying on parent- or caregiver-reported outcomes, particularly for sleep and behavioral measures, where assessor blinding was unclear or not reported. Accordingly, Adib et al. (2019) [19] and Ansari et al. (2021; sleep) [20] were rated as having a high overall risk of bias, while Ansari et al. (2021; kata) [21] and Zhao et al. (2024) [23] were judged as having some concerns, mainly due to incomplete reporting of allocation procedures and assessor blinding. Missing outcome data and selective reporting were not the major contributors to bias; however, small sample sizes reduced the precision of effect estimates. Overall, although randomized trials provided the strongest available evidence, confidence in the findings was limited by methodological and reporting constraints (Figure 4). 

**Figure 4:**
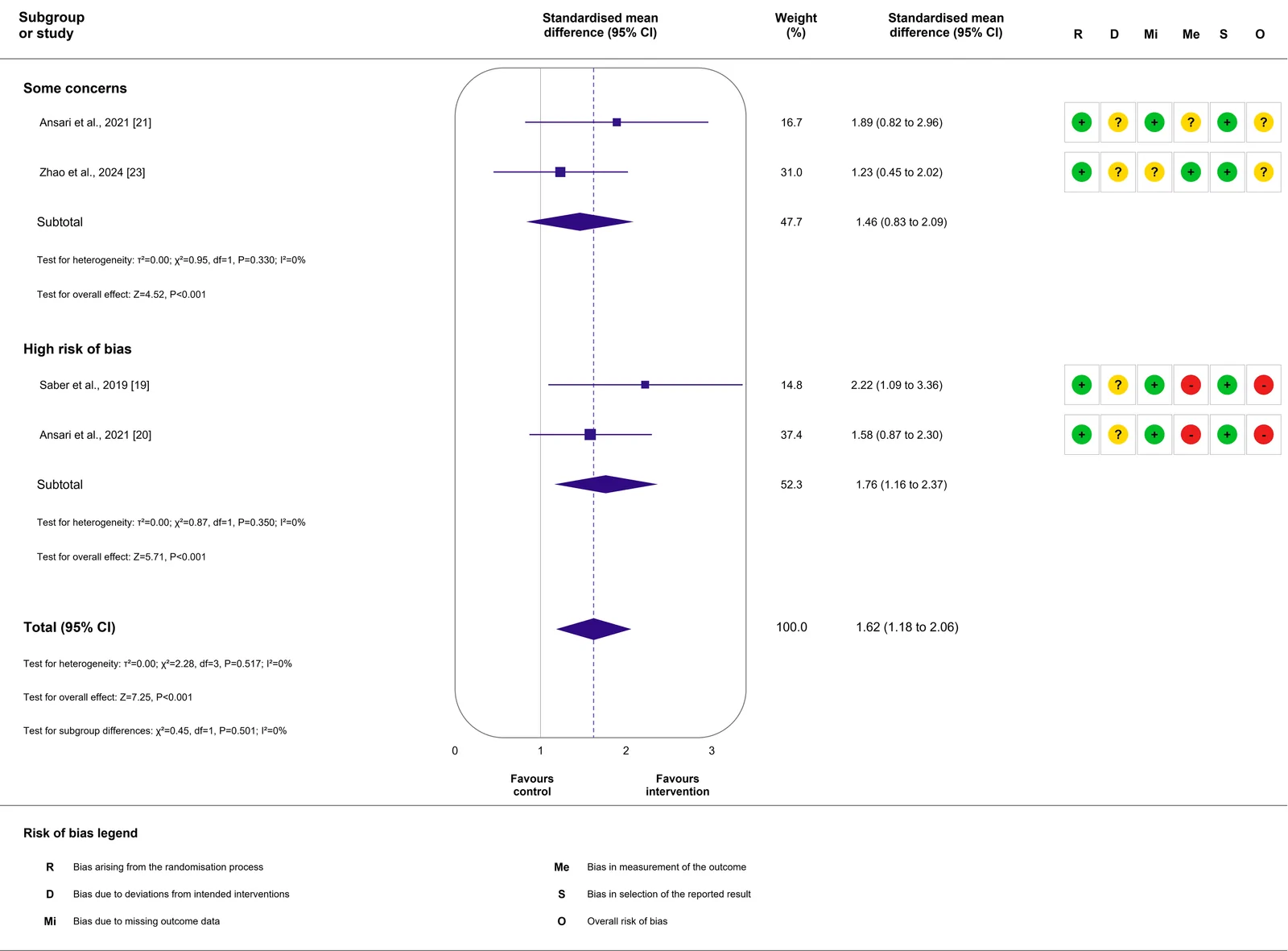
Forest plot stratified by overall risk of bias using RoB 2 for RCTs Studies were stratified according to the overall RoB 2 judgment. Effect sizes are presented as Hedges’ g with 95% confidence intervals and are coded so that positive values favor aquatic intervention. Green (+) indicates low risk of bias, yellow (?) indicates some concerns, and red (−) indicates high risk of bias RoB 2: Cochrane risk-of-bias tool for randomized trials; RCT: randomized controlled trial

Non-randomized controlled and quasi-experimental studies: Three non-randomized, quasi-experimental, controlled studies were assessed using the ROBINS-I tool. Overall, these studies demonstrated moderate to serious risk of bias, primarily due to confounding and participant selection. Caputo et al. (2018) and Zanobini and Solari (2019) were judged to be at serious risk of bias, as non-random factors, including geographical feasibility and a matched-control design, with incomplete follow-up reporting in the latter, influenced group allocation. Faraji et al. (2023) were rated as having a moderate risk of bias. However, residual concerns remained regarding group comparability due to a quasi-experimental design and recruitment across different clinical settings. As with the randomized studies, blinding of participants and providers was not feasible, introducing potential performance bias; however, additional concerns regarding confounding factors (e.g., baseline differences, intervention access, family motivation, and concurrent therapies) were more prominent in these studies. Detection bias was also noted, particularly when outcomes were subjective, and assessor blinding was not reported (Figure 5).

**Figure 5:**
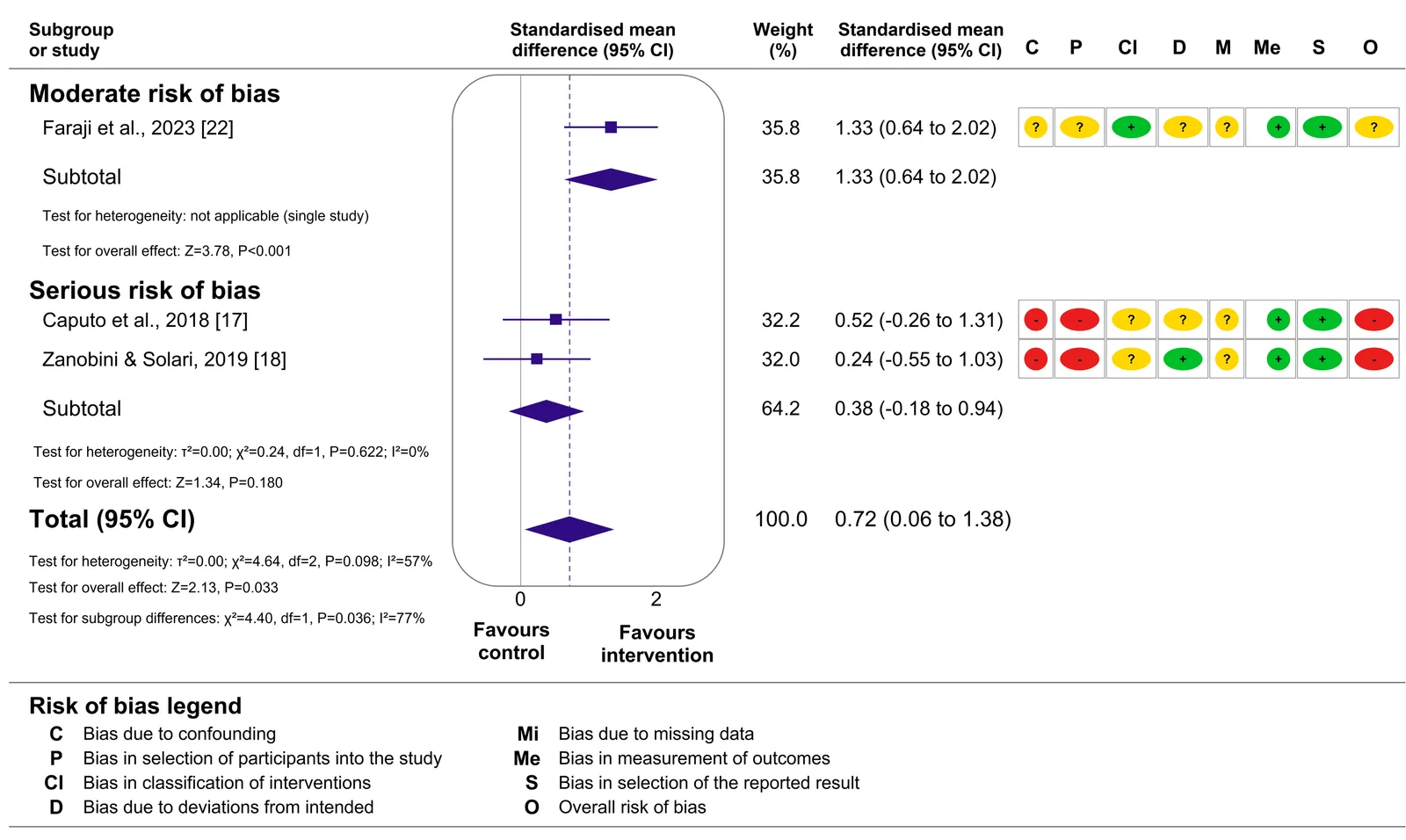
Forest plot stratified by overall risk of bias using ROBINS-I for Non-RCTs and Quasi-Experimental Studies This figure was created by the authors. Studies were stratified according to the overall ROBINS-I judgment. Effect sizes are presented as Hedges’ g with 95% confidence intervals and were coded so that positive values favor aquatic intervention. Green (+) indicates low risk of bias, yellow (?) indicates moderate risk/some concerns, and red (−) indicates serious or high risk of bias ROBINS-I: Risk of Bias in Non-randomized Studies - of Interventions

Overall, no study included in the quantitative synthesis was free of bias concerns. Although randomized trials provided relatively stronger evidence, findings from both randomized and non-randomized studies should be interpreted with caution, given study design limitations, small sample sizes, and incomplete reporting.

Discussion

This systematic review and meta-analysis synthesized evidence on aquatic interventions for children and adolescents with autism spectrum disorder (ASD), integrating quantitative findings from controlled studies with a complementary narrative synthesis. Overall, aquatic interventions were associated with beneficial effects across multiple domains, with the most consistent improvements observed in aquatic skills and water safety, motor and balance performance, sleep, and executive function. In contrast, social, behavioral, and adaptive outcomes demonstrated favorable yet statistically nonsignificant effects, likely reflecting the complexity and distal nature of these outcomes.

Aquatic interventions should not be conceptualized merely as swimming instruction but rather as a multidimensional therapeutic modality. The aquatic environment uniquely combines task-specific motor learning, graded physical activity, sensory modulation, and structured therapist-child interaction. The physical properties of water, including buoyancy, hydrostatic pressure, and viscosity, facilitate postural control, proprioceptive feedback, and safe movement practice, thereby reducing fear of injury and enhancing engagement [13,17,23]. These characteristics are particularly relevant for children with ASD, who often present with motor coordination challenges, sensory-processing differences, and restricted participation in physical activities [22].

Consistent with earlier studies, aquatic skills and water safety emerged as the most robust outcomes. This is expected because these skills are directly targeted and represent proximal treatment effects. Previous studies have consistently reported improvements in aquatic competence, including floating, breath control, and propulsion, following structured aquatic programs [26,27,28]. These findings reinforce that children with ASD can acquire water-related skills through individualized, structured interventions. Importantly, these improvements have significant clinical and safety implications, as children with ASD are at an increased risk of drowning [14,15]. Thus, gains in water competency extend beyond motor learning to support functional independence and safety, as emphasized in prior research.

Similarly, the observed improvements in motor and balance outcomes are consistent with previous evidence demonstrating that aquatic interventions can effectively address motor impairments in ASD. Children with ASD frequently present with deficits in coordination, balance, and motor planning [6,22], and the present findings are in line with studies reporting improvements in balance, strength, and physical fitness following aquatic therapy [21,22,24]. These findings support the hypothesis that the aquatic environment provides a unique therapeutic context in which buoyancy reduces gravitational load. At the same time, water resistance facilitates progressive strengthening and motor learning [13].

However, some studies have shown comparable benefits from structured land-based interventions [21], suggesting that improvements may be driven not only by the aquatic medium but also by the intensity, structure, and repetition of training. Aquatic interventions should therefore be considered within a broader range of therapeutic physical activity options.

The findings on sleep outcomes are also supported by prior research, although the evidence base remains limited. Studies such as Adib et al. [19] and Oriel et al. [30] have reported improvements in sleep latency and duration following aquatic exercise, consistent with the favorable effects observed in this review. These effects are biologically plausible and may reflect the combined influence of increased physical activity, sensory regulation, and structured routines, all of which are known to impact sleep in children with ASD [11,31]. Nevertheless, variability across studies and reliance on parent-reported measures suggest that these findings should be interpreted with caution, as highlighted in earlier research.

Preliminary improvements in executive function observed in this review are consistent with emerging evidence linking physical activity to cognitive outcomes in ASD. Faraji et al. and Zhao et al. [22,23] reported improvements in inhibitory control and cognitive flexibility, which align with broader meta-analytic findings demonstrating the positive effects of exercise on executive function in this population [34,35]. However, the limited number of studies and heterogeneity in outcome measures restrict the strength of these conclusions. These findings suggest potential neurocognitive benefits, although more rigorous evidence is needed.

In contrast, social, behavioral, and adaptive outcomes showed a favorable, but not statistically significant pattern, consistent with mixed findings reported in earlier studies. Previous research has highlighted variability in social outcomes depending on intervention design and outcome measurement [17,18]. For example, while some studies reported improvements in adaptive behavior and emotional regulation [17], others found limited effects on formal social communication measures [28,32]. These findings suggest that social and adaptive improvements are less direct and more dependent on the incorporation of explicit therapeutic strategies. Furthermore, social gains in children with ASD are often context-dependent, and improvements observed within the aquatic setting may not generalize to other environments without targeted interventions and extended exposure. This interpretation is consistent with the broader physical activity literature, which indicates that exercise alone may enhance readiness for interaction but does not necessarily yield generalized gains in social communication [36].

The current findings also align with emerging evidence on the multisystem nature of aquatic interventions, which integrate physical, sensory, and psychosocial components. Previous studies have suggested that aquatic therapy may influence outcomes through neurobiological and physiological pathways, including modulation of inflammatory markers and neurotrophic factors such as brain-derived neurotrophic factor (BDNF) [20,23]. These mechanisms are consistent with broader exercise literature, which highlights the role of physical activity in enhancing neuroplasticity and regulating physiological systems relevant to ASD [37,38,39]. In addition, studies have reported improvements in quality of life, participation, and caregiver satisfaction, emphasizing the importance of considering functional and family-centered outcomes alongside clinical measures [29,40].

In summary, this review supports aquatic interventions as an adjunctive therapeutic approach for children and adolescents with ASD, particularly for improving motor skills, water safety, and participation in physical activity. The findings are largely consistent with previous studies while providing additional quantitative support for domain-specific effects.

Clinical implications

The findings support aquatic intervention as a promising adjunctive therapy for children and adolescents with ASD, particularly when the clinical goals include water competency, motor skills, balance, sleep regulation, and participation in meaningful physical activity. Aquatic programs may be especially valuable for children who are attracted to water, have difficulty tolerating land-based exercise, require sensory input, or need structured opportunities for safe movement exploration. However, aquatic therapy should not be presented as a universal treatment for core ASD symptoms. Its effects appear domain-specific and dependent on intervention design.

Clinically, programs should be individualized and structured, with attention to safety, visual support, predictable routines, graded exposure, sensory preferences, communication strategies, and caregiver goals. For social/adaptive outcomes, interventionists should intentionally embed social communication goals such as turn-taking, imitation, peer interaction, joint attention, manding, waiting, and shared play. For executive-function outcomes, cognitively enriched aquatic activities may be required. For sleep outcomes, programs should monitor session timing, intensity, sensory response, and bedtime routines. For motor outcomes, progressive challenge and sufficient intensity are needed to move beyond water adjustment toward measurable gains in balance and fitness.

Limitations

The findings should be interpreted in light of several limitations. First, the limited number of controlled studies meant that most domain-specific meta-analyses included only two studies. As a result, heterogeneity estimates were unstable, and publication-bias testing was not appropriate. Second, studies varied substantially in design, intervention duration, frequency, intensity, therapeutic framework, comparator condition, and outcome measures. Third, blinding of participants and therapists was not feasible, and several outcomes relied on parent- or caregiver-reported measures, increasing the risk of expectation effects. Fourth, some non-randomized studies were vulnerable to confounding and selection bias. Fifth, the evidence base included small samples, and many studies did not provide sufficient information on autism severity, intellectual skills, sensory profile, medication use, concurrent therapies, or intervention fidelity. Finally, formal risk-of-bias tools were not applied to studies included only in the narrative synthesis; therefore, findings from these studies were considered supportive and should be interpreted cautiously.

Future research directions

Future research should prioritize adequately powered randomized controlled trials with transparent allocation procedures, blinded outcome assessment where feasible, standardized reporting of intervention fidelity, and longer-term follow-up. Trials should distinguish between aquatic intervention models, such as swimming instruction, Halliwick-based therapy, aquatic occupational therapy, multisystem aquatic therapy, sensory-enhanced aquatics, and aquatic exercise training, rather than treating all water-based interventions as equivalent. Intervention dose, intensity, therapist qualifications, adult-to-child ratio, and safety procedures should be reported in detail. Future studies should also examine moderators of response, including age, autism severity, intellectual ability, sensory profile, baseline motor skills, sleep disturbance profile, and prior water exposure. Finally, mechanistic studies combining behavioral outcomes with biomarkers, actigraphy, physiological arousal measures, and neurocognitive tasks may clarify whether aquatic intervention operates primarily through physical fitness, sensory regulation, neuroplasticity, emotional security, social mediation, or a combination of these pathways.

## Conclusions

Aquatic interventions appear to offer a promising, meaningful, and clinically relevant therapeutic approach for children and adolescents with ASD. The strongest evidence supports improvements in aquatic skills, water safety, and motor/balance performance, while sleep and executive-function outcomes show promising but still preliminary benefits. Social, behavioral, and adaptive outcomes appear favorable but uncertain, likely because these changes depend on whether social communication and functional generalization are explicitly targeted. The aquatic environment may be best understood as an enriched therapeutic context rather than a single intervention modality: it provides physical support, resistance, sensory input, emotional security, structured learning, and opportunities for participation. When these elements are intentionally tailored to the child’s needs and therapeutic goals, aquatic intervention may contribute not only to motor and safety outcomes but also to broader developmental, behavioral, and participation-related benefits.
